# Potassium iodide enhances the antimicrobial activity of plasma-activated water

**DOI:** 10.1016/j.bioflm.2025.100313

**Published:** 2025-08-22

**Authors:** Laura A. McClenaghan, Thomas P. Thompson, Akash Shambharkar, Ross M. Duncan, Paula Bourke, Timofey Skvortsov, Brendan F. Gilmore

**Affiliations:** a Biofilm Research Group, School of Pharmacy, Queen's University Belfast, Medical Biology Centre, 97 Lisburn Road, Belfast, BT9 7BL, UK; b Plasma Research Group, School of Biosystems and Food Engineering, University College Dublin, Dublin 4, Ireland

**Keywords:** Cold plasma, Plasma-activated water, Potassium iodide, Biofilms, Hypoiodous acid, Food pathogens, *Salmonella*

## Abstract

Plasma-activated water (PAW) is a promising disinfection strategy that generates a complex mixture of reactive oxygen and nitrogen species (ROS/RNS), including hydrogen peroxide (H_2_O_2_), nitrate (NO_3_^−^), and transient oxidants, in an acidic aqueous environment. These reactive species contribute to both immediate and extended antimicrobial activity. This study investigates how the addition of low concentrations (<100 μM) of potassium iodide (KI) enhances the bactericidal properties of spark-generated PAW by enabling the in-situ generation of reactive iodine species (RIS), particularly hypoiodous acid (HIO), under acidic conditions.

KI addition (10–100 μM) led to a counterintuitive, dose-dependent increase in H_2_O_2_ concentrations, from ∼1.2 mM in PAW alone to ∼1.8 mM at 30 μM KI, possibly due to iodine-mediated catalytic effects or reduced H_2_O_2_ degradation. NO_3_^−^ levels also increased by ∼17 % with increasing KI. Equivalent concentrations of H_2_O_2_ + KI failed to replicate the rapid antimicrobial activity observed in PAW + KI, which achieved complete inactivation of *Escherichia coli* and *Listeria monocytogenes* planktonic cells within 3 min, compared to over 10 min for PAW alone, indicating the involvement of additional reactive species in KI-enhanced antimicrobial activity of PAW. However, *Salmonella enterica planktonic cells* exhibited only partial inactivation even with KI, indicating species-specific tolerance under these conditions. 24h biofilms of *L. monocytogenes* and *E. coli* were eradicated with PAW + KI in 10 min, whereas *S. enterica* showed only a 2-log reduction.

Scavenger assays revealed that both longer-lived species (H_2_O_2_) and shorter-lived oxidants such as singlet oxygen are essential for this enhanced killing, while ozone and superoxide appeared dispensable. These findings support a multi-step antimicrobial mechanism: (1) plasma treatment creates a low pH, H_2_O_2_-rich solution; (2) iodide is oxidised to RIS such as I_3_^−^ and HIO; (3) additional PAW-derived oxidants potentiate RIS chemistry; and (4) unionised HIO diffuses across bacterial membranes to induce oxidative damage.

PAW-KI remained stable for at least 14 days at 4 °C, with sustained RIS activity and minimal loss of H_2_O_2_ or NO_3_^−^, suggesting preserved antimicrobial capacity over time. The antimicrobial mechanism likely proceeds through a four-step pathway: plasma-mediated generation of H_2_O_2_ and NO_3_^−^; oxidation of I^−^ to I_2_ and HIO; potentiation of RIS via PAW-derived ROS/RNS; and subsequent microbial inactivation via membrane damage.

Together, these results demonstrate that PAW + KI forms a powerful, in situ RIS-generating system, offering a residue-minimising and environmentally sustainable disinfection platform. Its rapid action, scalability, and reliance on only air, water, electricity, and GRAS-listed KI make it an attractive intervention for food safety, clinical disinfection, and decentralised sanitation settings.

## Highlights

• Combining KI with PAW forms reactive iodine species (e.g., I₂, HOI) *in situ* under acidic conditions.
• PAW + KI achieves rapid inactivation (7-log reduction) of *Escherichia coli* and *Listeria monocytogenes* within 3 min.
• KI enhances PAW production of hydrogen peroxide (H_2_O_2_) and nitrate (NO_3_^−^), increasing overall oxidative stress.
• Antimicrobial action is driven by singlet oxygen and hypoiodous acid, with species-specific effects.
• PAW+KI is shelf-stable, using only air, water, and KI, for a scalable, preservative-free, eco-friendly disinfection method.

## 1 Introduction

Ensuring microbial safety remains a major challenge in global food production, as persistent pathogens such as *Escherichia coli*, *Salmonella enterica*, and *Listeria monocytogenes* pose significant risks to public health [1]. These pathogens not only have the capacity to cause severe illness but also exhibit high tolerance to traditional sanitising agents by forming biofilms - structured microbial communities encased in a self-produced extracellular matrix (ECM) that shields them from disinfectants and cleaning procedures [2].

Non-thermal, atmospheric pressure or ‘cold’ plasma technologies, particularly those capable of generating plasma-activated water (PAW), have emerged as a promising non-antibiotic alternative strategy for microbial inactivation, with potential environmental advantages depending on energy sourcing and application design. The breadth of plasma modalities, antimicrobial mechanisms, and device considerations across applications have been discussed recently [3]. Importantly, as outlined in a recent review [4], PAW has been proposed as a non-toxic alternative to conventional chemical disinfectants and may align with sustainability goals in the food industry. PAW is generated by exposing water to a cold plasma discharge for a defined period of time, during which reactive species from the plasma translocate into the aqueous phase via interaction at the air-liquid interface and subsequent diffusion into the bulk liquid, producing an array of reactive oxygen (ROS) and nitrogen species (RNS) in solution [5]. These reactive species contribute to PAW's potent antimicrobial activity by inducing oxidative damage to bacterial membranes, proteins, and metabolic pathways [6].

Numerous studies have validated the efficacy of PAW in reducing microbial bioburden across a range of food products including poultry [7], beef [[8], [9], [10], [11]], seafood [12,13], eggs [14], tofu [15], and fresh produce [[16], [17], [18]], often without compromising sensory qualities. PAW has also been used to degrade pesticide residues from food products [19,20].

However, despite these benefits, the efficacy of PAW against microbial biofilms and highly biocide-tolerant bacterial strains remains limited in some cases - potentially due to the presence of only sublethal concentrations of long-lived reactive species, such as hydrogen peroxide (H_2_O_2_), nitrite (NO_2_^−^), and nitrate (NO_3_^−^), which alone may be insufficient to overcome biofilm-associated tolerance mechanisms [5,21].

This highlights the importance of either optimising PAW or ROS/RNS composition or incorporating it into processes with complementary antimicrobial agents/biocides for enhanced efficacy. To enhance the potency of PAW, several studies have explored combining PAW with agents such as tartaric acid [18], and propylparabens [16]. For example, Qian et al., demonstrated a 2-log reduction in *Salmonella enteritidis* burden on beef slices when enhanced with lactic acid compared to PAW alone [10]. While these approaches can enhance antimicrobial activity, consideration must also be given to ensure that PAW's environmental compatibility is maintained or that such additives to PAW, intended to increase antimicrobial efficacy, do not alter the organoleptic and flavour profiles of treated products.

In this study, we explore the use of potassium iodide (KI) as a green enhancer of PAW antimicrobial activity against several bacteria associated with food spoilage or food-borne infection outbreaks. Iodine-based compounds have well established potent antimicrobial effects, and prior work [22] has shown that plasma-generated H_2_O_2_ can oxidise iodide to triiodide (I_3_^−^), a known bactericidal species. This reaction also underpins a simple, colourimetric assay to quantify H_2_O_2_ [23], widely used across environmental and photocatalytic systems, though not yet routinely applied to PAW. However, despite this prior work, the specific chemical interplay between PAW-derived ROS/RNS and iodide ions under ambient, spark discharge conditions has not been thoroughly characterised. In particular, the formation, speciation, and stability of short-lived reactive iodine species (RIS) such as hypoiodous acid (HIO) remain poorly defined in plasma–liquid systems. This represents a notable gap in the current understanding of PAW enhancement strategies, which this study seeks to address. We hypothesise that PAW and KI forms a synergistic oxidative system, in which plasma derived ROS/RNS drive the in-situ generation of antimicrobial iodine species system, enhancing killing of both biofilm and planktonic bacteria.

We investigate the antimicrobial efficacy of PAW + KI against major foodborne pathogens in both planktonic and biofilm states, evaluate the chemical stability of the system during refrigerated storage, and conduct ROS/RNS scavenger assays to identify the reactive species responsible for bacterial killing. Together, these findings support PAW–KI synergy as a novel disinfection strategy with the potential to enhance food safety and reduce reliance on conventional chemical biocides, while warranting further investigation into its environmental implications.

This approach combines GRAS-listed components (potassium iodide and plasma-activated water) and generates reactive species in situ, thereby minimising chemical storage, reducing harmful residues, and potentially replacing halogenated disinfectants such as chlorine or quaternary ammonium compounds, which may raise environmental or regulatory concerns.

## 2 Materials and methods

### 2.1 Chemical reagents

Potassium Iodide (99.99 %) was purchased from Fluorochem (Hadfield, UK). Polycarbonate disc coupons (0.5-inch x 0.15-inch) (RD128-PC) were purchased from BioSurface Technologies (Montana, USA).

### 2.2 Bacteria used in this study

The following food pathogens were used in the study: *E. coli* NCTC 12241*, S. enterica* DSM 17058, and *L. monocytogenes* NCTC 10357. All strains were inoculated in Mueller-Hinton broth (MHB) and grown overnight with shaking at 37 °C. For all experiments, the optical density (OD_600_) was adjusted accordingly for each bacterium and diluted to achieve a starting inoculum corresponding to 1 × 10^8^ CFU/mL.

### 2.3 Generation of PAW

**Fig. 1:**
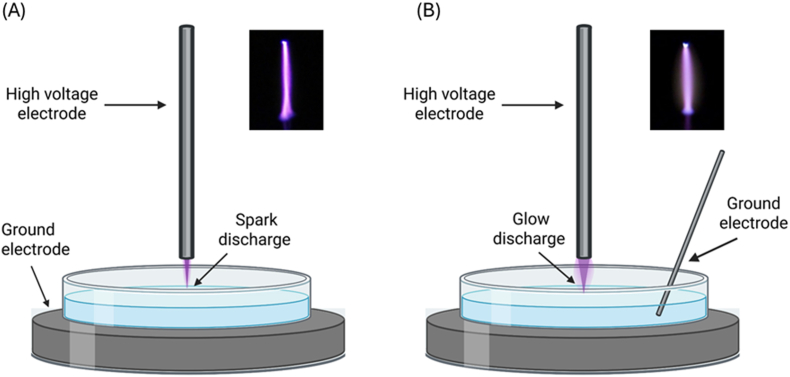
Setup of Spark (A) and Glow (B) discharge to make PAW. The plasma from the different electrical set-up generates a visually different plume. Created in BioRender. Mcclenaghan, L. (2025) https://BioRender.com/d57a154.

Two types of electrical discharge configurations were set up to generate the indirect PAW as described previously by Ref. [24,25]. In both setups, a stainless-steel needle served as the high voltage electrode, positioned perpendicular to the water surface at a fixed 5 mm distance, with 10 mL of deionised water (DIW) or DIW + KI. For Spark discharge, the petri dish was placed on a grounded stainless-steel plate (Fig. 1A), using 4 kV and ∼25 kHz. For Glow discharge, a submerged ground electrode rod was used (Fig. 1B) with 2 kV applied. Both discharges were operated in open air for 10 min.

### 2.4 Quantification of ROS/RNS

The concentration of H_2_O_2_ in PAW was measured using titanium (IV) oxysulphate, which forms a yellow pertitanic acid complex that absorbs strongly at (405 nm). A 10 μL titanium sulphate solution was added to 100 μL of PAW or standard concentrations of H_2_O_2,_ and absorbance at 405 nm was measured after a 10 min incubation and converted to H_2_O_2_ concentrations using a standard curve. NO_3_^−^ concentration was determined using the NO_3_^−^ Spectraquant Assay (Sigma Aldrich, UK) with sulfamic acid pre-treatment to eliminate NO_2_^−^ interference. Standards and PAW samples were measured at 340 nm after a 10 min incubation away from light. NO_2_^−^ formation was measured using the Griess test (Sigma Aldrich), where samples were diluted tenfold, reacted with sulphanilamide and naphthyl-ethylenediamine, and incubated in the dark for 30 min before absorbance was measured at 548 nm. The pH was recorded using a calibrated pH meter (Hanna, Edge), with three replicates for each measurement.

### 2.5 Planktonic and biofilm susceptibility

Antimicrobial susceptibility testing was carried out initially in planktonic cultures. The OD of *E. coli, S. enterica* or *L. monocytogenes* was adjusted to approximately 0.1 (OD_600_) and 100 μL of this bacterial suspension was added to 900 μL PAW + KI treatment and PAW control such that a standard final inoculum density of 1 × 10^7^ CFU/mL was used in each assay (n = 3). This elevated bioburden was chosen to simulate contamination levels that may occur in high-load environments, such as post-biofilm disruption or surface residue in food processing settings, and to enable accurate resolution of bactericidal activity. At specific timepoints (0, 1, 3, and 5 min) 20 μL of the test solution was removed and added into 180 μL PBS in a 96-well microtitre plate. The bacterial suspensions were serially diluted and 10 μL was plated onto Mueller-Hinton agar (MHA) plates in triplicate. The MHA plates were then incubated overnight at 37 °C and the surviving colonies were counted after 24 h.

Biofilms were grown on polycarbonate coupons by inoculating each coupon in a 24-well plate with 1 mL of an OD adjusted bacterial culture of 0.1 (OD_600_). Coupons were incubated for 24 h at 37 °C to allow biofilms to form. Before treatment, biofilms were rinsed with PBS to remove any loosely attached planktonic cells. Then, 1 mL of each PAW + KI (50 μM or 100 μM) and PAW (as control) were then added to the wells for specific treatment times (1, 3, 5 and 10 min). Following treatment of the biofilm for the set time, the PAW was removed, and the biofilm was rinsed with PBS. Finally, 1 mL of fresh PBS was added, and the 24-well plate was sonicated for 20 min in a Branson 3510 sonic bath. The supernatant was then serially diluted and 10 μL was plated onto MHA plates in triplicate. The MHA plates were then incubated overnight at 37 °C and colonies were counted after 24 h.

### 2.6 LIVE/DEAD staining

Biofilms of *E. coli* and *S. enterica* were grown on polycarbonate coupons and treated with PAW or PAW supplemented with 100 μM KI for 0, 3, or 5 min. After treatment, the biofilms were gently rinsed with PBS and stained using the LIVE/DEAD™ BacLight™ Bacterial Viability Kit (Thermo Fisher) according to the manufacturer's instructions. Biofilms were imaged using a Leica TCS SP8 confocal laser scanning microscope (CLSM) with excitation/emission settings of 488/500–540 nm for SYTO 9 (live cells) and 561/590–650 nm for propidium iodide (dead cells). Z-stacks were collected across the biofilm thickness and processed using maximum intensity projection to visualise structural and viability changes.

### 2.7 Scavenger assay

Chemical scavengers were used to specifically quench reactive species generated in the PAW and PAW + KI solutions to investigate their roles in antimicrobial activity against planktonic bacteria. Scavenger types and concentrations were selected based on previous studies and included sodium pyruvate (10 mM) for H_2_O_2_, l-histidine (20 μM) for singlet oxygen and other ROS, uric acid (100 μM) for ozone [26] and Tiron (20 mM) for superoxide ion [27].

### 2.8 pH adjusted antimicrobial testing of PAW + KI

The pH of the PAW + KI was adjusted post plasma exposure to assess whether ionised iodine was the active antimicrobial agent, due to the pKa of HIO being approximately 10.5. Batches of the PAW +50 μM KI was adjusted to pH of 5, 7.5 and 10.5 and planktonic kill curves were carried out as previously mentioned to examine whether a shift toward ionised iodine species corresponded with diminished antimicrobial activity. We note that this pH adjustment may also influence other reactive components in PAW, and this limitation is discussed accordingly.

### 2.9 Stability of PAW + KI

A 14-day stability study was carried out to monitor the stability of H_2_O_2_, NO_3_^−^ and the pH in PAW and PAW + KI. Three batches of each PAW and PAW + KI solution were generated and stored at 4 °C. H_2_O_2_, NO_3_^−^ and the pH were measured on days 0, 1, 7 and 14. The antimicrobial activity of PAW + KI against planktonic bacteria was also evaluated after 7 days of storage at 4 °C.

### 2.10 Statistical analysis

**Fig. 2:**
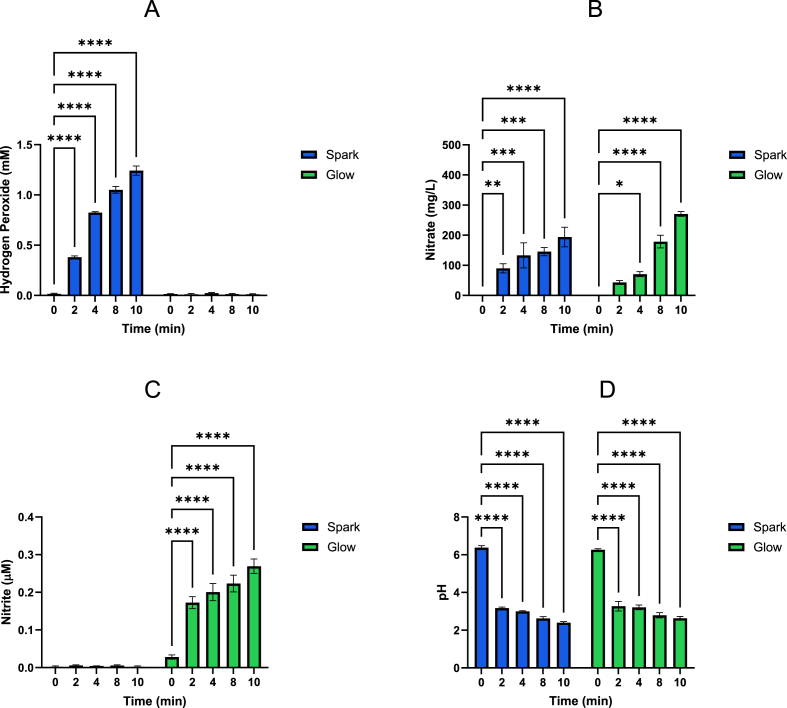
Generation of H_2_O_2_ (A), NO_3_^−^ (B), NO_2_^−^ (C) and the change in pH (D) of deionised water following treatment with either a Spark or Glow discharge. The Spark PAW contains H_2_O_2_ and NO_3_^−^, while the Glow PAW contains NO_3_^−^ and NO_2_^−^ (n = 3).

**Fig. 3:**
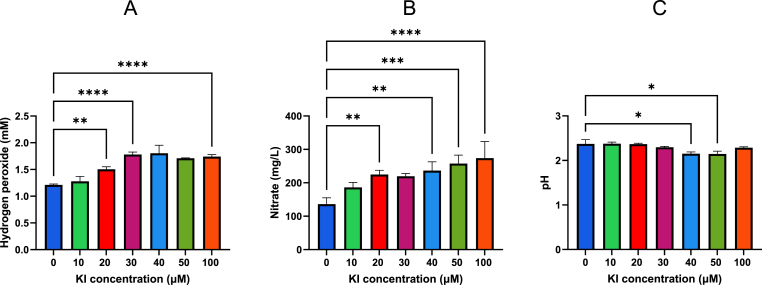
The H_2_O_2_ (A) and NO_3_^−^ (B) measurements and pH changes (C) for PAW and PAW + KI. The addition of KI caused an increase in both H_2_O_2_ and NO_3_^−^ species, whereas the pH remained relatively the same throughout**.** (n = 3).

**Fig. 4:**
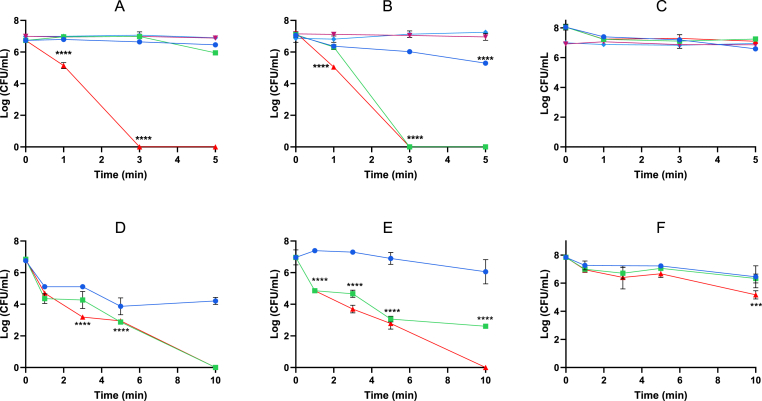
The antimicrobial efficacy of PAW and PAW + KI against *E. coli* (A), *L. monocytogenes* (B) and *S. enterica* (C) in planktonic phenotype. Spark PAW only , PAW +10 μM  PAW +50 μM , 10 μM KI only , and 50 μM KI only . The antimicrobial efficacy of PAW and PAW + KI against *E. coli* (D), *L. monocytogenes* (E) and *S. enterica* (F) 24 h biofilms grown on polycarbonate coupons. Spark PAW only , PAW +50 μM , and PAW +100 μM . (n = 3).

**Fig. 5:**
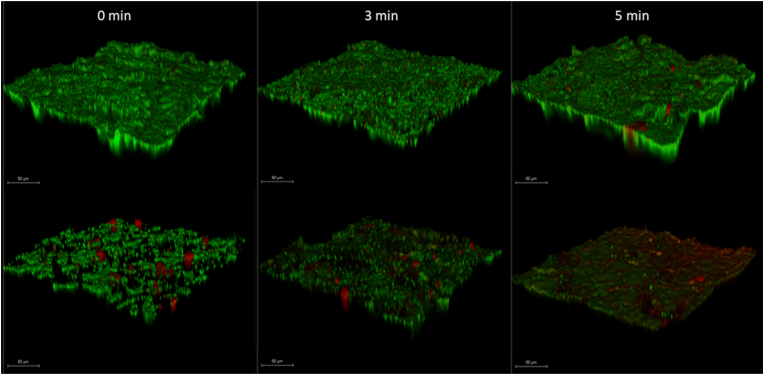
CLSM images of *S. enterica* (top row) and *E. coli* (bottom row) biofilms following PAW + 100 μM KI treatment for 0-, 3-, and 5-min. Biofilms were stained with LIVE/DEAD stain, where green indicates viable cells and red indicates membrane-compromised or dead cells, Images show increasing cell death over time, particularly in *E. coli*. Scale bars = 50 μm.

For data presented in Fig. 2, Fig. 4, Fig. 5 the average means and standard deviation from biological triplicates were compared using a two-way ANOVA and Bonferroni's test. For Fig. 3, Fig. 7, a one-way ANOVA, with Dunnett's post-hoc test against the untreated control were used. P values of ∗ (P ≤ 0.05), ∗∗ (P ≤ 0.01), ∗∗∗ (P ≤ 0.001), and ∗∗∗∗ (P ≤ 0.0001) indicate statistical significance. GraphPad Prism 10.4.1 was used for all analyses.

## 3 Results

### 3.1 Measurement of ROS/RNS in PAW

Spark- and Glow-generated PAW differ markedly in their reactive species profiles, as previously reported [24,25]. Spark PAW contained high levels of H_2_O_2_ (1.357 mM after 10 min), along with NO_3_^−^, while glow PAW produced mainly NO_3_^−^ (228.26 mg/L) and NO_2_^−^ (223.80 μM), with negligible H_2_O_2_ (Fig. 2). Despite similar acidification kinetics - both treatments reduced pH rapidly to ∼2.5 - only Spark PAW offered the ROS composition necessary for effective iodide oxidation. Based on this, Spark-generated PAW was selected for all downstream experiments with KI.

### 3.2 Measurement of ROS/RNS in spark-generated PAW + KI

Spark-generated PAW was prepared with increasing concentrations of KI (10–100 μM) and analysed for H_2_O_2_, NO_3_^−^, and pH.

Compared to PAW alone (1.2 mM H_2_O_2_), the addition of KI led to a dose-dependent increase in apparent oxidising capacity, reaching ∼1.8 mM by 30 μM KI. Although iodide can react with H_2_O_2_ under acidic conditions, the increase observed suggests that plasma-generated H_2_O_2_ outpaces its consumption. KI may also help preserve H_2_O_2_ by interfering with its breakdown via reactive radicals. NO_3_^−^ levels also increased with KI, while NO_2_^−^ remained undetectable. The pH of PAW (∼2.37) showed a modest but statistically significant decrease at 40–50 μM KI (pH ∼2.15), suggesting that KI may influence buffering capacity or promote formation of acidic by-products. Together, these results indicate that KI alters PAW's ROS/RNS profile in a concentration-dependent manner while maintaining the acidic environment required for plasma-derived reactivity.

### 3.3 Antimicrobial activity of PAW and PAW + KI

The bactericidal efficacy of PAW and PAW + KI was assessed against *E. coli*, *L. monocytogenes,* and *S. enterica* in both planktonic and biofilm modes of growth (Fig. 4). PAW alone achieved a 2-log reduction in *L. monocytogenes* but had no measurable effect on *E. coli* or *S. enterica.* When supplemented with 10 μM KI, PAW achieved complete inactivation of *L. monocytogenes* within 3 min. A higher concentration (50 μM KI) was required to fully eradicate *E. coli* within the same timeframe. However, *S. enterica* remained partially tolerant under all tested conditions, with no complete killing observed even at 50 μM KI. This enhanced tolerance may relate to previous reports showing reduced susceptibility of *S. enterica* to iodine-based disinfectants in environmental and agricultural settings [28].

PAW and PAW + KI was then tested against 24 h biofilms of *E. coli*, *L. monocytogenes,* and *S. enterica* on polycarbonate coupons. Complete biofilm eradication was achieved with PAW +100 μM KI after 10 min for *L. monocytogenes*, and with both 50 μM and 100 μM KI for *E. coli*. In contrast, *S. enterica* biofilms showed only a 2-log reduction after 10 min with 100 μM KI. These findings indicate enhanced tolerance of *S. enterica* biofilms and demonstrate the superior efficacy of the combined PAW + KI system. A comparative summary of KI concentrations, ROS/RNS concentrations, and antimicrobial outcomes are provided in Table 1.

**Table 1:** Comparative summary of the physicochemical properties (pH, H_2_O_2_ concentration, NO_3_^−^ levels) and antimicrobial efficacy against planktonic and biofilm forms for Spark PAW and PAW + KI (10, 50, and 100 μM). “–” indicates not tested (n = 3).

	Spark PAW	PAW + KI (10 μM)	PAW + KI (50 μM)	PAW + KI (100 μM)
pH	2.37	2.35	2.15	2.27
**H_2_O_2_ (mM)**	1.2	1.27	1.70	1.74
**NO_3_^−^ (mg/L)**	136	186.2	257.4	273.9

	**Planktonic log-reduction in CFU/mL (5-min treatment time)**			

***E. coli***	0.28	0.79	6.74	–
***L. monocytogenes***	1.69	7.06	7.22	–
***S. enterica***	1.46	0.8	0.95	–

	**Biofilm log-reduction in CFU/mL (10-min treatment time)**			

***E. coli***	2.63	–	6.75	6.84
***L. monocytogenes***	0.91	–	4.36	6.97
***S. enterica***	1.39	–	1.5	2.67

### 3.4 LIVE/DEAD staining

Fluorescence microscopy using LIVE/DEAD staining supports the CFU/mL data, showing greater susceptibility of *E. coli* biofilms to PAW + KI compared to *S. enterica*. After 5 min of exposure to PAW +100 μM KI, *E. coli* biofilms showed widespread red staining, consistent with a 4-log reduction in viability. Some green signal remained, as min was chosen to capture early-stage killing prior to complete eradication at 10 min. In contrast, *S. enterica* biofilms remained largely green, suggesting a higher proportion of intact, viable cells, matching the modest 1-log reduction observed. Together, these findings highlight the enhanced tolerance of *S. enterica* biofilms and confirm the greater efficacy of PAW + KI against *E. coli* under identical treatment conditions.

### 3.5 Scavengers assay

**Fig. 6:**
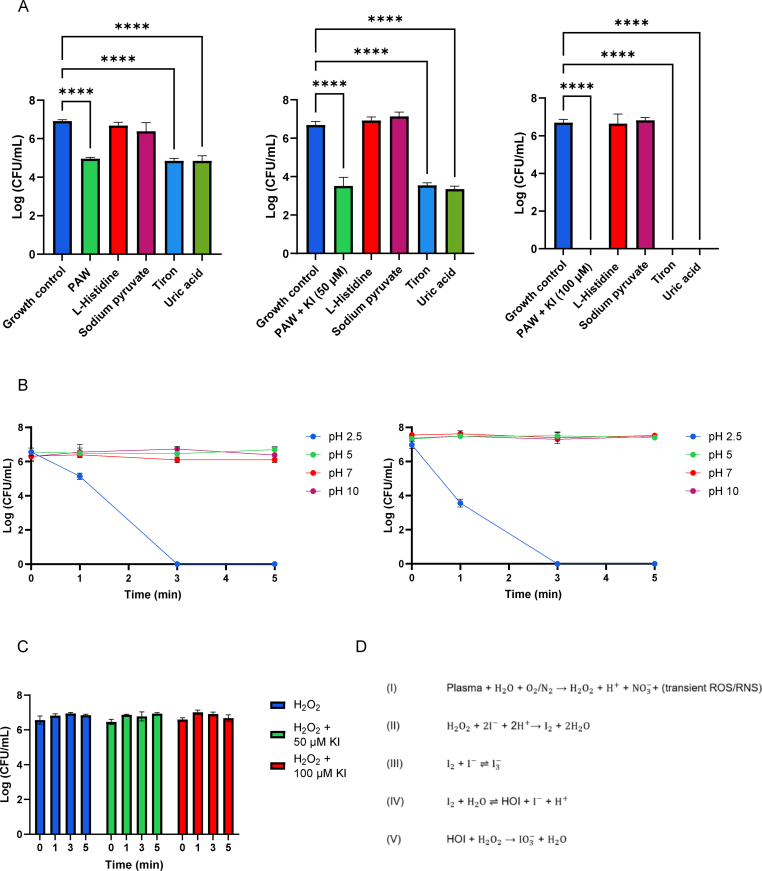
Impact of chemical scavengers on the antimicrobial activity of PAW, PAW + KI (50 μM) and PAW + KI (100 μM) with scavengers targeting H_2_O_2_ (sodium pyruvate), singlet oxygen and other longer-living ROS (l-histidine), ozone (uric acid), superoxide anions (O_2_^−^) (Tiron) (A). pH adjusted PAW + KI (50 μM) planktonic *E. coli* kill curve. The pH of the PAW + KI was adjusted post plasma exposure to inadvertently assess whether ionised iodine was the active antimicrobial agent, due to the pKa of HIO being approximately 10.5. No reduction in *E. coli* was observed when the pH was adjusted to pH of 5, 7 or 10, suggesting that unionised HIO is the main antimicrobial agent in PAW + KI. (n = 3). (B). Assessing the antimicrobial activity of H_2_O_2_ (1.2 mM) and of H_2_O_2_ (1.2 mM) in combination with either 50 μM or 100 μM KI in planktonic culture. The presence of KI was shown not to enhance the antimicrobial effect of H_2_O_2_, suggesting that it is the unique combination of reactive species within PAW which is responsible for this effect (n = 3). (C). Proposed reaction mechanism of the PAW and KI system (D). **(I)** Cold plasma exposure of water in the presence of ambient air (O_2_/N_2_) generates H_2_O_2_, protons (H^+^), NO_3_^−^, and a variety of transient ROS/RNS. **(II)** In the acidic environment of PAW, I^−^ from KI are oxidised by H_2_O_2_ to I_2_. **(III)** I_2_ reacts with excess I^−^ to form triiodide (I_3_^−^). **(IV)** I_2_ can also hydrolyse to form HIO, a potent antimicrobial species. **(V)** Over time, HIO may be further oxidised by H_2_O_2_ to iodate (IO_3_^−^), an inert end-product.

To determine the reactive species responsible for the antimicrobial activity of PAW and PAW + KI, scavenger assays were performed, as previously described, with *E. coli*, which exhibited the greatest sensitivity to PAW in prior tests. Specific chemical scavengers were selected to neutralise key reactive species: 20 mM Tiron (superoxide), 10 mM sodium pyruvate (H_2_O_2_ and other ROS), 20 mM l-histidine (singlet oxygen and other longer-lived ROS), and 100 μM uric acid (ozone) [25]. In both PAW and PAW + KI treatments (Fig. 6), the addition of sodium pyruvate appeared to negate the antimicrobial activity, lead to bacterial counts (∼10^7^ CFU/mL) after treatment that were statistically indistinguishable from the untreated control. This indicates that scavenging ROS primarily H_2_O_2_ results in loss of antimicrobial activity highlighting the critical role of ROS in microbicidal activity. Furthermore, l-histidine produced a similar effect, suggesting an additional contribution from singlet oxygen or other persistent ROS. In contrast, tiron and uric acid had negligible impact on bacterial survival in the PAW + KI treatment, implying that ozone and superoxide anions are unlikely to be a major contributor to the observed bactericidal activity.

### 3.6 Stability of PAW + KI

**Fig. 7:**
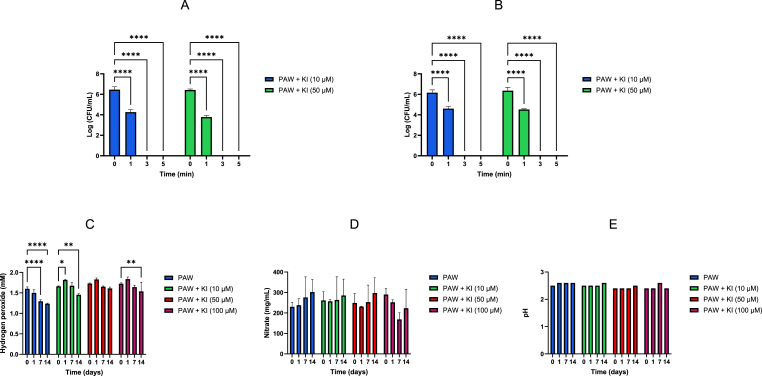
Antimicrobial activity of PAW + KI after storage at 4 °C for 7 days against planktonic cells of *E. coli* (A) and *L. monocytogenes* (B). The stability of H_2_O_2_ (C) and NO_3_^−^ (D) measurements and pH changes (E) for PAW and PAW + KI stored at 4 °C. The addition of KI caused an increase in both H_2_O_2_ and NO_3_^−^ species, whereas the pH remained unchanged throughout (n = 3).

The stability of the H_2_O_2_ and NO_3_^−^ species, as well as pH was monitored at specific timepoints (0, 1, 7 and 14 days). PAW alone showed a gradual decrease in H_2_O_2_ over time with a significant decrease after 7 days (P < 0.0001). PAW +10 μM KI and PAW +100 μM KI showed a significant decrease in H_2_O_2_ only after 14 days of storage, while PAW +50 μM KI showed no decrease in H_2_O_2_ at any time point (Fig. 7C). No significant difference was observed in NO_3_^−^ concentrations or pH for any groups after the longest time point, suggest these properties are stable in both PAW and PAW + KI solutions.

The antimicrobial activity of the PAW + KI was also tested after storage for 7 days at 4 °C. Both the PAW +10 μM KI and PAW +50 μM KI retained their antibacterial activity against *E. coli* and *L. monocytogenes* (Fig. 7A–B).

## 4 Discussion

This study demonstrates that combining PAW and KI generates an antimicrobial system with significantly greater activity than PAW or H_2_O_2_ alone. Building on the findings of Ke et al. [22], who showed that KI can be oxidised to the bactericidal triiodide (I_3_^−^) in the presence of helium plasma, we show that a similar synergistic effect can be achieved with Spark PAW, which contains millimolar concentrations of H_2_O_2_. Ke et al. previously confirmed this reaction is catalase-sensitive, identifying H_2_O_2_ as the principal oxidant in their system [22]. Although we did not directly measure iodine species, our scavenger assays and increased levels of H_2_O_2_ suggest a similar H_2_O_2_-mediated mechanism.

While the classic H_2_O_2_ + KI reaction produces I_3_^−^ and HIO, our results suggest that this reaction alone cannot explain the full extent of PAW + KI's enhanced antimicrobial activity. For example, PAW + KI achieved complete inactivation of *E. coli* and *L. monocytogenes* within 3 min, whereas matched concentrations of H_2_O_2_ (1.2 mM) + KI did not achieve comparable killing (Fig. 6C). This suggests that PAW provides a favourable microenvironment, characterised by low pH, elevated nitrate, and short-lived ROS, that uniquely supports the formation and activation of RIS.

Although iodide–H_2_O_2_ interactions are well-known in classical chemistry, famously exemplified in the iodide-catalysed 'Elephant's Toothpaste' reaction, there remains a distinct lack of mechanistic data on how plasma-derived ROS/RNS, particularly short-lived species such as peroxynitrite and singlet oxygen, interact with iodide under ambient-air Spark discharge conditions. To our knowledge, this is the first study to explore this specific chemical interplay. We propose that PAW offers a unique physicochemical environment, distinct from purely chemical or biological systems, that supports novel iodine-based oxidative reactions driven by short-lived plasma-derived species, a chemical interplay not previously explored in KI–plasma systems under ambient-air discharge conditions.

Notably, KI addition increased the H_2_O_2_ concentrations from ∼1.2 mM in PAW alone to ∼1.8 mM at 30 μM KI (Fig. 3A), potentially due to iodine-mediated catalytic effects or reduced H_2_O_2_ decomposition. The concentrations of NO_3_^−^ also increased with increasing KI concentrations, while NO_2_^−^ remained below the assay limit of detection. This may indicate that iodide influences nitrogen speciation indirectly, though the mechanism remains unclear. The PAW pH remained consistent (∼2.37–∼2.15 at 40–50 μM KI), sustaining conditions known to promote RIS generation. Additionally, some studies have shown that nitrite and H_2_O_2_ can react synergistically in acidified PAW to generate peroxynitrite (ONOO^−^), a highly reactive species that contributes significantly to bacterial inactivation in plasma-treated liquids [29,30]. While we did not directly measure ONOO^−^, the presence of nitrite in Glow PAW and rising NO_3_^−^ levels in Spark PAW + KI suggest that reactive nitrogen intermediates may also influence iodine chemistry and antimicrobial activity.

Among the possible RIS generated, unionised HIO appears to be the dominant species under acidic conditions, while the relative contributions of RIS (I_3_^−^, I_2_, HIO) may shift over time or with pH, warranting further time-resolved monitoring. HIO is known to have potent antimicrobial activity due to its neutral charge, membrane permeability, and reactivity with biomolecules. Our pH-adjusted experiments support this hypothesis: neutralisation of PAW + KI to pH 5, 7.5, or 10.5 significantly suppressed antimicrobial activity (Fig. 6B). This trend is consistent with a pH-driven shift away from the unionised, membrane-permeable form of HIO, which predominates at acidic pH below its pKa (∼10.5). However, we recognise that adjusting the pH of PAW post-plasma exposure may also alter the concentration and stability of other reactive oxygen (ROS) and nitrogen species (RNS), as well as key physicochemical properties such as oxidation–reduction potential and conductivity. These factors may independently or synergistically influence antimicrobial activity and should be considered when interpreting these results. Therefore, while our findings are consistent with HIO ionisation contributing to reduced efficacy, we acknowledge that broader changes in PAW chemistry may also play a role.

The oxidation of I^−^ in PAW can yield a range of species including I_2_, HIO, I_3_^−^, and eventually IO_3_^−^. Importantly, iodate (IO_3_^−^) is chemically inert and non-bactericidal [31], and is considered a terminal oxidation product in other systems. However, we observed no loss of antimicrobial activity in ours, as RIS activity remained high for at least 14 days when stored at 4 °C, with no significant drop in H_2_O_2_ or NO_3_^−^ concentrations after 14 days (Fig. 7). These findings suggest that the sustained antimicrobial potential of the PAW + KI system is maintained by a reservoir of long-lived oxidative species, including hydrogen peroxide, nitrate, and possibly stable iodine species such as I_3_^−^ or residual I_2_.

To understand this mechanism further, we conducted scavenger assays using *E. coli* (Fig. 6 A). Sodium pyruvate (a H_2_O_2_ quencher) and l-histidine (a singlet oxygen scavenger and related species) both abolished activities, returning CFU/mL counts to baseline (∼10^7^ CFU/mL), highlighting the essential role of H_2_O_2_. However, the enhanced antimicrobial effect of PAW + KI over matched H_2_O_2_ + KI also implies a synergistic role for short-lived ROS and RNS uniquely generated in this system.

In contrast, uric acid and tiron had no effect, suggesting that ozone and superoxide play minimal roles in this system. These results are consistent with prior plasma studies showing that non-H_2_O_2_ oxidants (e.g., peracetic acid, singlet oxygen) significantly contribute to antimicrobial activity even in catalase-treated systems [32].

In addition to the effects of reactive species generated by PAW and iodide oxidation, species-specific differences were observed. In planktonic models, PAW +10 μM KI was sufficient to eradicate *L. monocytogenes*, while *E. coli* required 50 μM KI for complete inactivation within 3 min (Fig. 4A–C). In biofilm models, complete eradication of *E. coli* and *L. monocytogenes* required 100 μM KI and 10 min exposure (Fig. 4D–E). However, *S. enterica* remained tolerant under all tested conditions, achieving only a 2-log reduction even at the highest KI concentration and longest exposure. These trends were reflected in LIVE/DEAD CLSM imaging (Fig. 5): *E. coli* biofilms exhibited intense red fluorescence (dead cells) after 5 min treatment, while *S. enterica* biofilms remained predominantly green (viable). This tolerance may be rooted in differential oxidative defence capacities. Unlike *E. coli*, which encodes a single alkyl hydroperoxide reductase (AhpC), *S. enterica* also encodes *alkyl* hydroperoxide reductase (AhpC), thiol peroxidase (Tpx), as well as multiple catalases [33]. Moreover, *S. enterica* biofilms often exhibit denser extracellular matrices and enhanced redox buffering, providing further protection against oxidative stress.

Unlike traditional iodophor antiseptics (e.g., povidone-iodine, Lugol's), which can be cytotoxic and leave persistent residues [34], PAW + KI generates short-lived RIS like HIO and I_3_^−^ that decompose rapidly. While not directly characterised in this study, the transient nature of RIS has been reported in related plasma-iodide systems [31] and may account for the low residue risk observed here, supporting the safe use of PAW + KI in food-processing applications.

Finally, the antimicrobial effect appears to be a result of four interlinked steps: (1) Spark plasma treatment generates a low pH, H_2_O_2_-rich solution; (2) iodide is rapidly oxidised to RIS (HIO, I_3_^−^); (3) additional reactive species generated in PAW - including singlet oxygen and RNS - potentiate RIS chemistry; and (4) unionised HIO and other RIS diffuse into cells and cause oxidative damage. While this mechanism is supported by chemical and antimicrobial data, key steps remain unverified. In particular, the cellular uptake of unionised HIO and its role in oxidative damage have not been directly demonstrated. This represents a key limitation of the current study. Future work should include membrane integrity assays (e.g., propidium iodide staining), intracellular reactive species detection (e.g., DCFH-DA), and electron microscopy to visualise cellular damage. Transcriptomic or proteomic analyses may further clarify stress response pathways and biomolecular targets of RIS under PAW + KI treatment. Recent studies have begun to explore these mechanistic dimensions in PAW-treated biofilms, including transcriptomic profiling and structural integrity assays [35]. Additionally, our group has demonstrated oxidative stress-mediated synergy in *P. aeruginosa* biofilms [36] and enhanced phage susceptibility in *P. mirabilis* following PAW pre-treatment [25], which together provide a framework for future mechanistic validation of PAW–RIS systems.

This system represents a scalable, non-toxic, residue-minimising disinfection approach generated using only air, water, electricity, and GRAS-listed KI. It is suitable for use in food processing, wound care, and decentralised sanitation, combining low toxicity with rapid on-demand generation. Moreover, it may be compatible with other antimicrobial strategies: recent work from our group demonstrated PAW significantly enhances phage-mediated killing in *Proteus mirabilis* biofilms [25], supporting further exploration of cold plasma combinatorial/synergistic biofilm control approaches.

## Author contributions

Conceptualization – BG, TPT

Data Curation – LMcC, RMD, AS

Formal Analysis – LMcC, TPT

Funding Acquisition – BG

Investigation – LMcC

Methodology – BG, LMcC

Project Administration – BG

Resources – BG, PB

Supervision – BG

Validation – LMcC, RMD, AS

Visualization – TPT, LMcC

Writing – Original Draft Preparation – LMcC, TPT, RMD

Writing – Review & Editing – LMcC, TPT, BG, PB, TS

All authors have read and approved the final version of the manuscript.

## Conflicts of Interest

All authors have no conflicts of interest to declare.

## Ethics Declaration

No animals, patient samples or human tissue were used in this research. No patient data was collected or analysed. All research involved in vitro laboratory experiments.

## Funding

Research reported in this publication was supported by the 10.13039/100000002National Institutes of Health (NIH) under award numbers RO1 AR076941 and the 10.13039/100017291Northern Ireland HSC Research & Development Division, Public Health Agency award STL/5350/17, as part of a Tripartite Grant to BG, and PB and by the 10.13039/100016337Department for the Economy, Northern Ireland, as part of a CAST Studentship Award with Linden Foods.

## Declaration of interest

The authors declare that they have no known competing financial interests or personal relationships that could have appeared to influence the work reported in this paper.

## Data Availability

All data generated or analysed during this study are included.
